# Supplementary material for: Genetic variation drives seasonal onset of hibernation in the 13-lined ground squirrel
Source: Commun Biol. 2019 Dec 20;2:478. doi: 10.1038/s42003-019-0719-5 (PMC6925185; doi:10.1038/s42003-019-0719-5)
Supplement: Supplementary file 1 — Supplementary Information [file 42003_2019_719_MOESM1_ESM.pdf]

Supplementary Figures

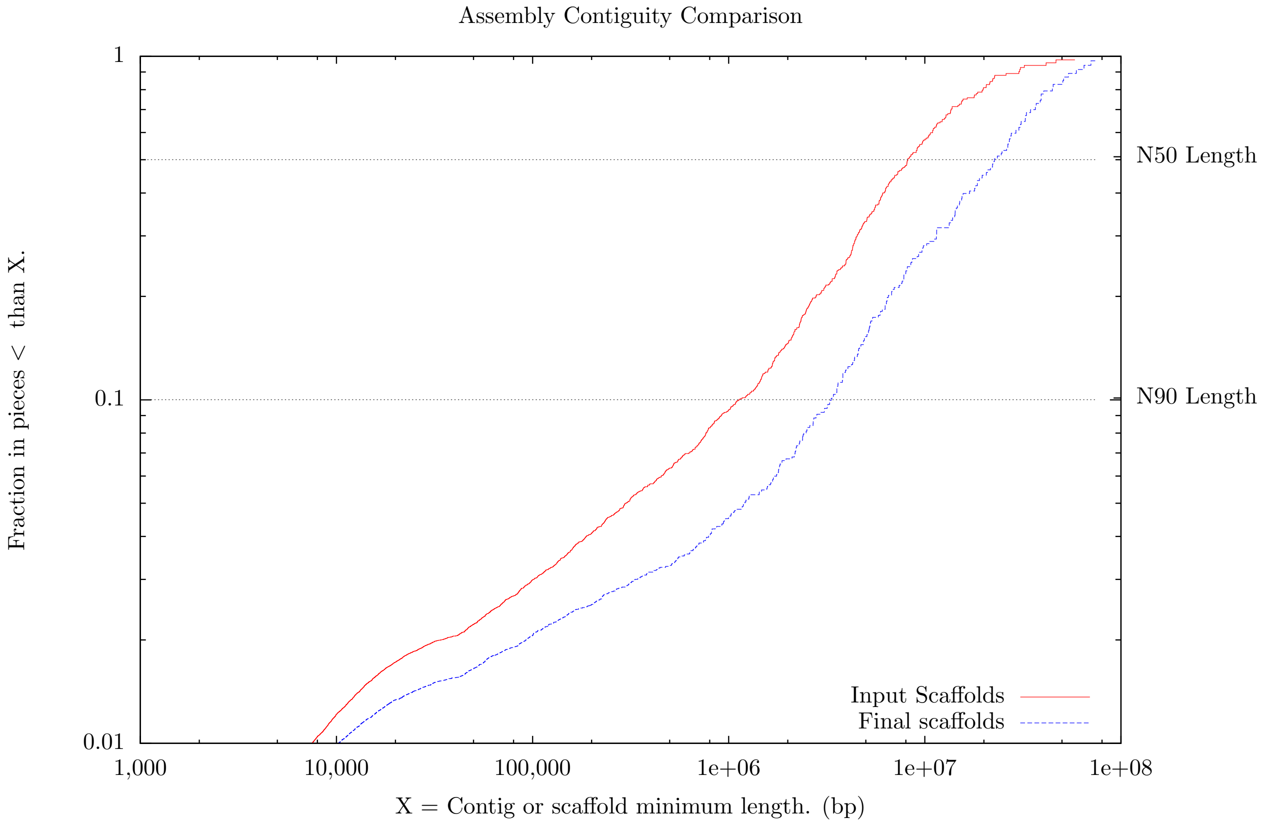

Supplementary Fig. 1. Comparison of the contiguity of the input assembly and the final HiRise scaffolds. Each curve shows the fraction of the total length of the assembly in scaffolds of a given length or smaller. The fraction of the assembly is indicated on the Y-axis and the scaffold length in basepairs is given on the X-axis. The two dashed lines mark the N50 and N90 lengths of each assembly. This plot excludes scaffolds less that 1 kb.

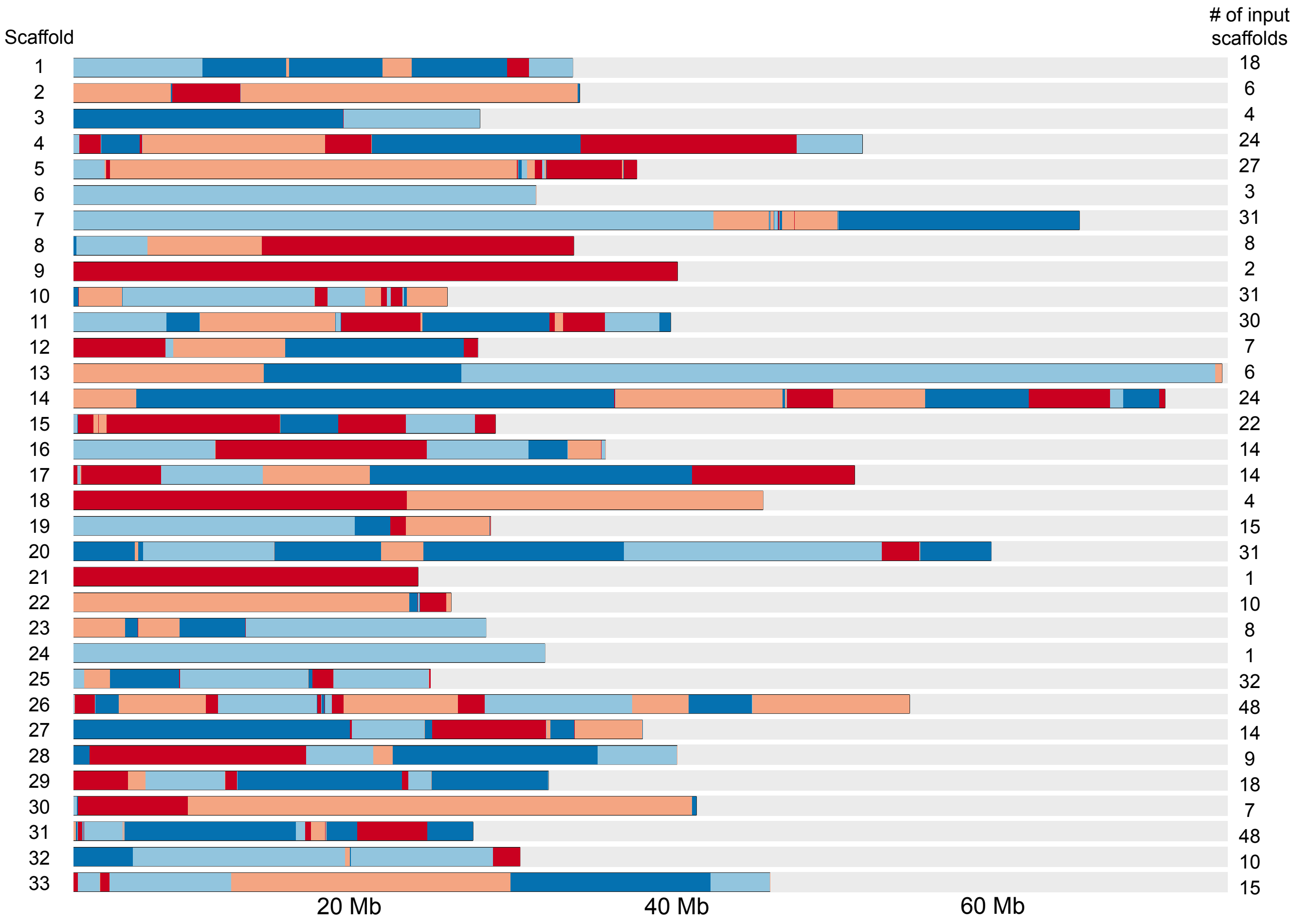

Supplementary Fig. 2. HiRise assembly improves the scaffold N50: 539 draft assembly scaffolds are reduced to 33. Each bar represents a HiRise scaffold. Each color within the bar represents a draft assembly scaffold. Note: colors are used to show placement of input scaffolds but are not specific to any one scaffold. Number of draft input scaffolds are listed on right.

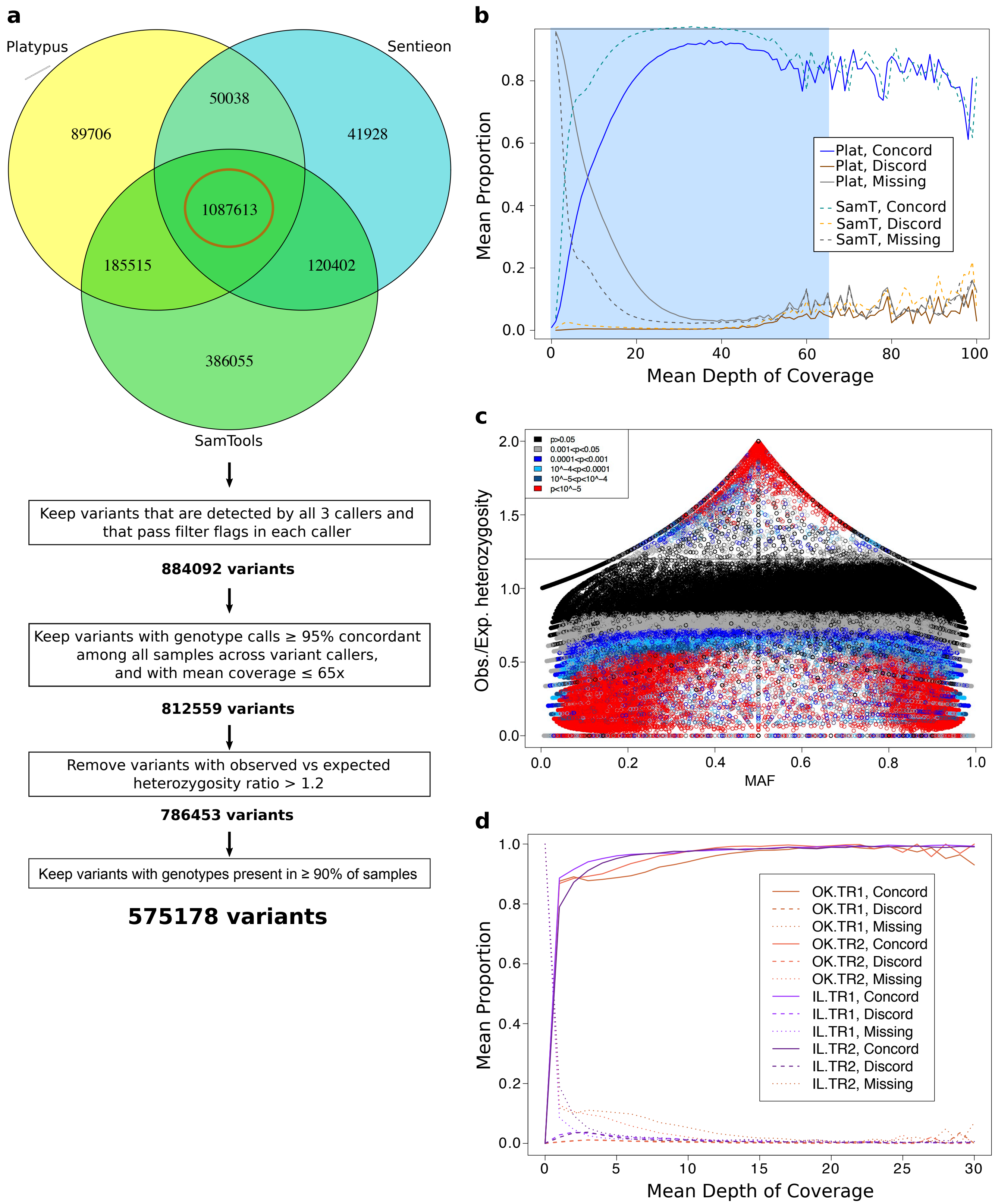
a
Platypus
Sentieon
SamTools
89706
50038
41928
1087613
185515
120402
386055
Keep variants that are detected by all 3 callers and that pass filter flags in each caller
884092 variants
Keep variants with genotype calls ≥ 95% concordant among all samples across variant callers, and with mean coverage ≤ 65x
812559 variants
Remove variants with observed vs expected heterozygosity ratio > 1.2
786453 variants
Keep variants with genotypes present in ≥ 90% of samples
575178 variants
b
Mean Proportion
Mean Depth of Coverage
Plat, Concord
Plat, Discord
Plat, Missing
SamT, Concord
SamT, Discord
SamT, Missing
c
Obs./Exp. heterozygosity
MAF
p>0.05
0.001<p<0.05
0.0001<p<0.001
10^-4<p<0.0001
10^-5<p<10^-4
p<10^-5
d
Mean Proportion
Mean Depth of Coverage
OK.TR1, Concord
OK.TR1, Discord
OK.TR1, Missing
OK.TR2, Concord
OK.TR2, Discord
OK.TR2, Missing
IL.TR1, Concord
IL.TR1, Discord
IL.TR1, Missing
IL.TR2, Concord
IL.TR2, Discord
IL.TR2, Missing

Supplementary Fig. 3. Variant calling pipeline and results. **a** The Venn diagram (top) shows the number of unique and shared variants detected by Platypus, Sentieon and SamTools variant callers. The flow chart beneath outlines the filtering strategy and the number of retained variants after each filtering step. **b** For the 884,092 variants detected by and passing filter flags in each variant caller, plot shows the mean proportion of variant calls that were concordant (Concord), discordant (Discord) or Missing between Sentieon and Platypus (Plat) and Sentieon and SamTools (SamT) among all samples as a function of coverage. Blue box highlights criterion range of ≤ 65x coverage. **c** The observed versus expected heterozygosity ratio of each variant, represented as an open circle and plotted by minor allele frequency (MAF). Those with a ratio >1.2, above the black horizontal line, were filtered from the dataset. Each variant is colored according to its Hardy-Weinberg equilibrium test statistic p-value bin listed in the legend. **d** For the 575,178 variants passing all filtering steps, the plot shows the mean proportion of variant calls that were concordant (Concord), discordant (Discord) or Missing between duplicate libraries of 4 samples (two samples from UW OshKosh, WI, "OK.TR1" and "OK.TR2"; and two samples from IL, "IL.TR1" and "IL.TR2") as a function of coverage.

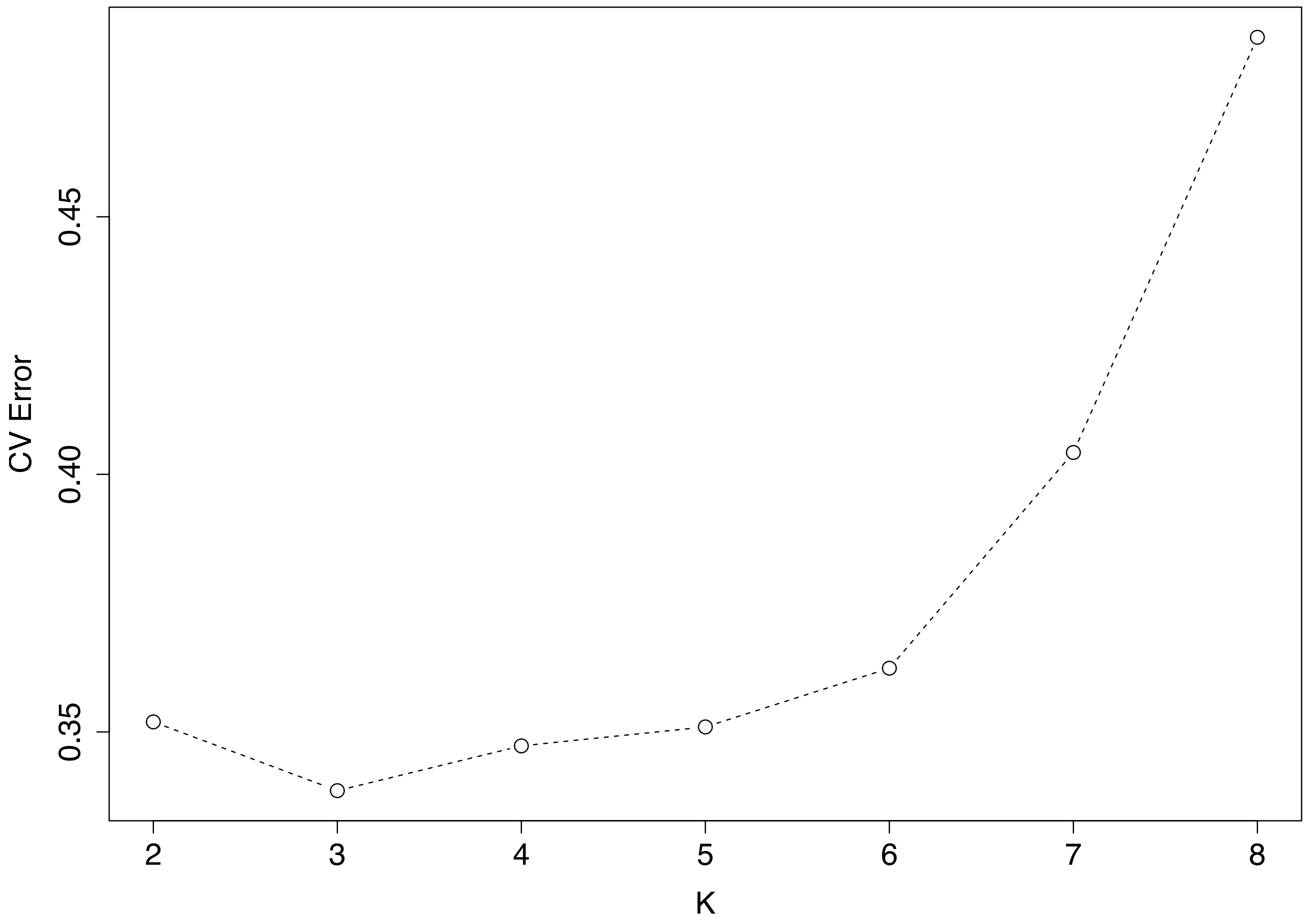

Supplementary Fig. 4. ADMIXTURE 5-fold cross-validation (CV) error for each value of *K*. Shown are the CV error values for *K*=2 through *K*=8.

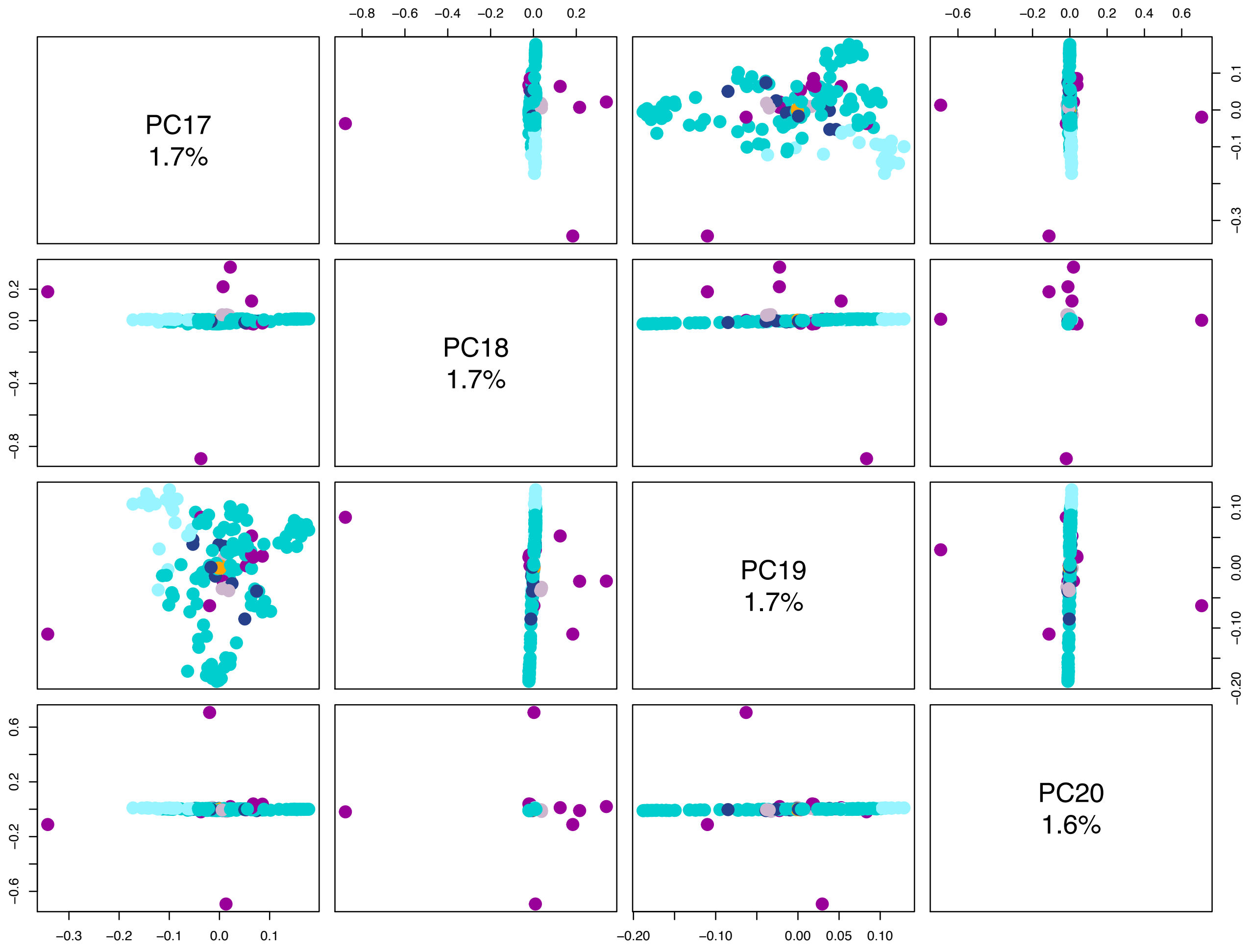

Supplementary Fig. 5. Principal components PC17 and PC19 of all 153 genotyped squirrels reveal population structure within the OshKosh subset of squirrels. Shading is the same as in Fig. 2a and 2c.

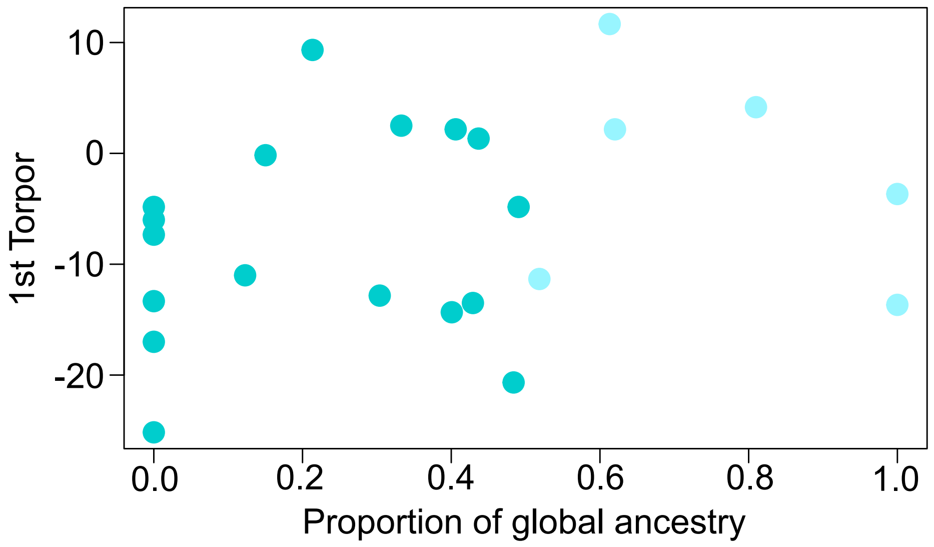

Supplementary Fig. 6. Proportion of global ancestry and timing of hibernation onset among the unrelated OshKosh subset of squirrels. Proportion of global ancestry is from the ADMIXTURE estimate for the *OK2* Oshkosh sub-population. Shading matches Fig. 2a and 2c. Hibernation onset (1st Torpor) is plotted as days ± from hibernaculum placement.

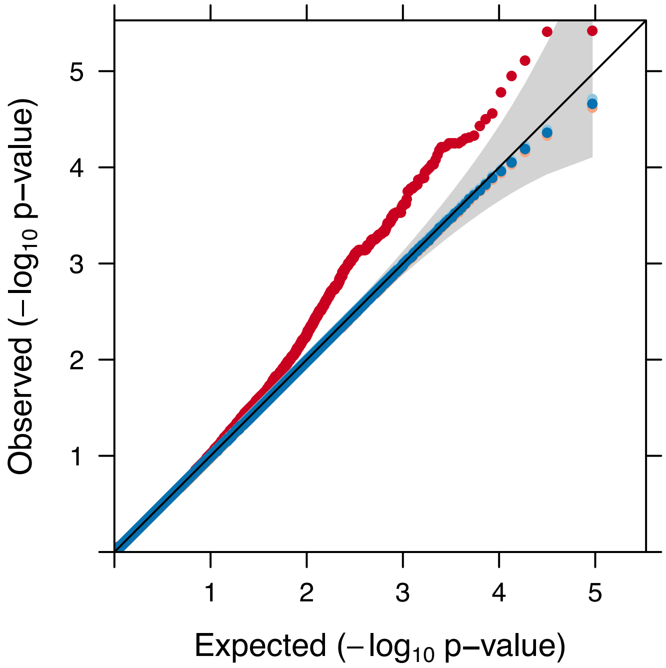

Supplementary Fig. 7. Q-Q plot of the GWAS log-transformed p-values using real and permuted datasets. Log-transformed p-values from the GWAS using the real dataset are plotted in red against their expected values. Median log-transformed p-values from 5,000 GWAS permutations are plotted in dark blue, with the upper and lower 97.5% confidence interval p-values plotted light blue and beige, respectively.

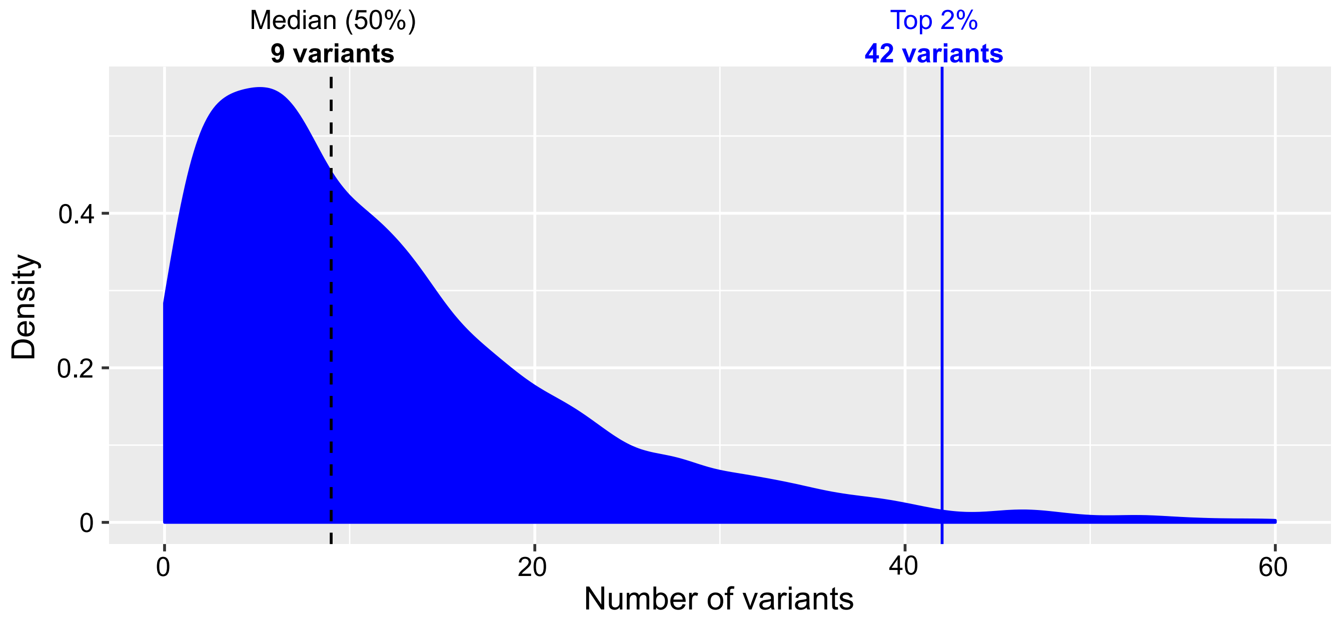

Supplementary Fig. 8. The empirical distribution in number of variants meeting suggestive significance. The density of variants meeting the exploratory p-value threshold of $p=2.13 \times 10^{-4}$ from 5,000 GWAS permutation tests are plotted. The dashed black vertical line denotes the median number of variants reaching the significance threshold. The solid blue vertical line marks the number of suggestively-associated variants observed in the real (non-permuted) dataset, demarking just the top two percent of this distribution, i.e., the top two percentile.

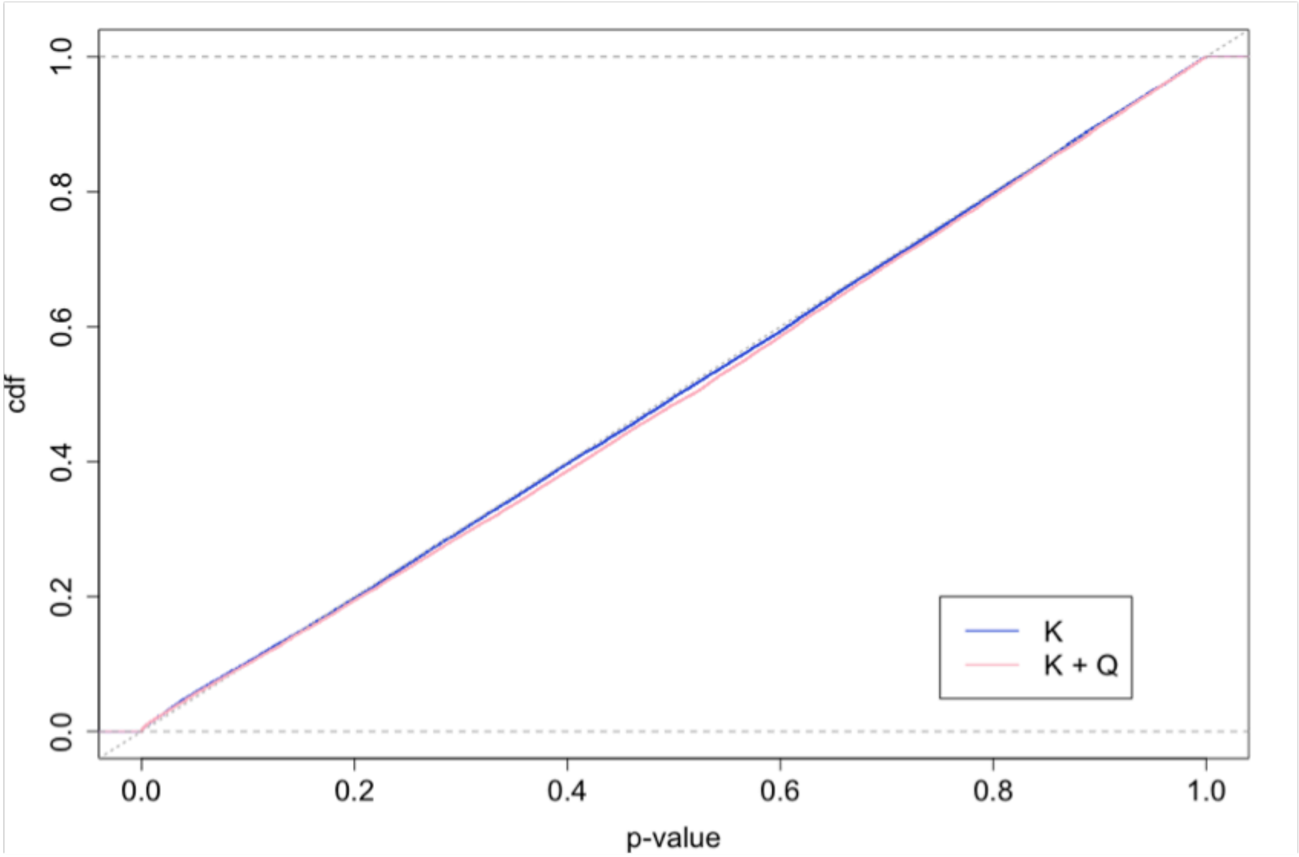

Supplementary Fig. 9. Cumulative Distribution of p-values from the hibernation onset GWAS. Blue curve shows distribution after correcting for population structure using only the genetic relatedness matrix (K) as a random effect in the linear mixed model. The pink curve shows distribution after correcting for population structure using genetic relatedness as a random effect and ancestry assignments from the ADMIXTURE algorithm as a fixed effect in the linear mixed model (K+Q). Diagonal gray dotted line denotes the curve of the expected distribution under the null model. cdf, cumulative distribution function.

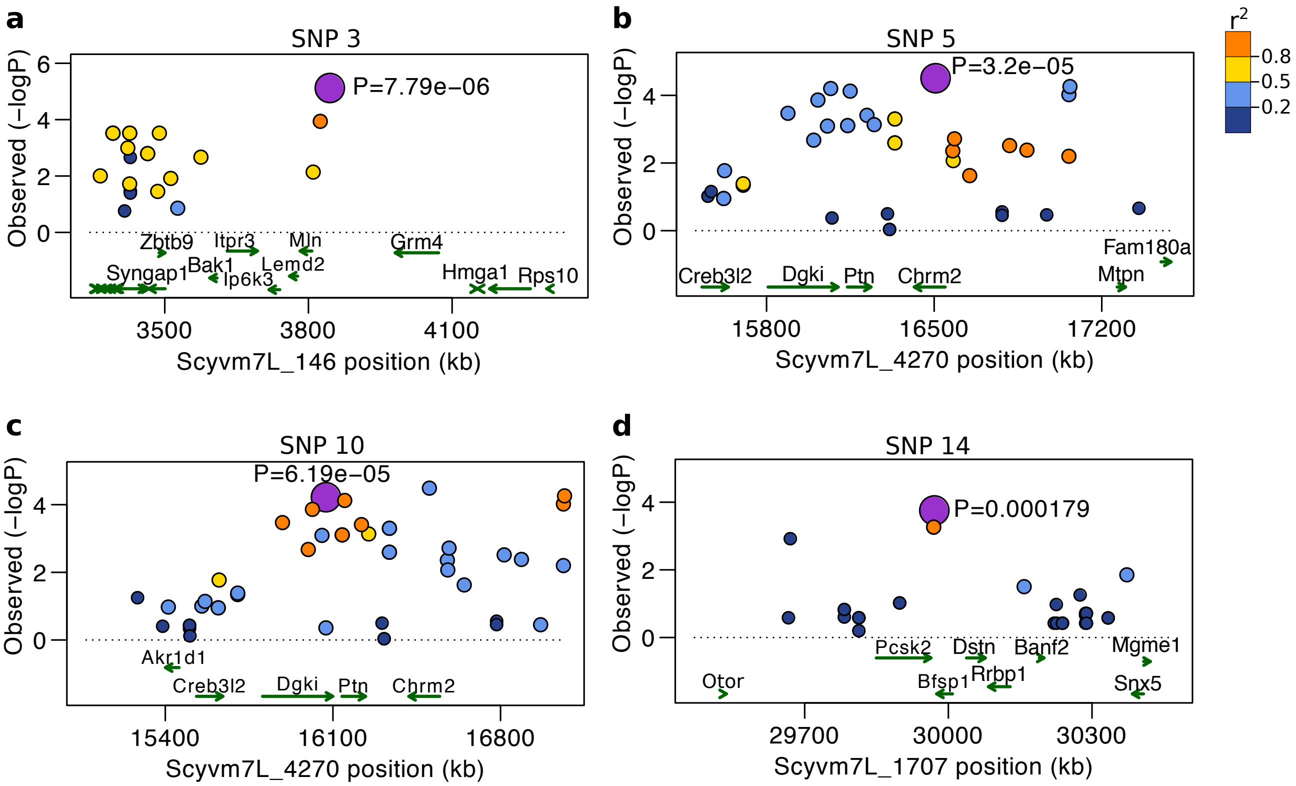

Supplementary Fig. 10. Regional Manhattan plots show locations of selected GWAS-suggestive variants in proximity to nearest genes. Each plot is centered on one of the suggestive variants, which are labeled on top by number (see Fig. 3a and Table 3) and shaded purple. Other variants within region are colored by LD value (r^2) in relation to the significant SNP. Genes are shown below as green arrows and labeled by gene symbol. **a** SNP 3 is nearest *MLN.* **b** SNP 5 is located nearest *CHRM2*. **c** SNP 10 is located nearest *DGKI* and *PTN*. **d** SNP 14 is between *PCSK2* and *BFSP1*.

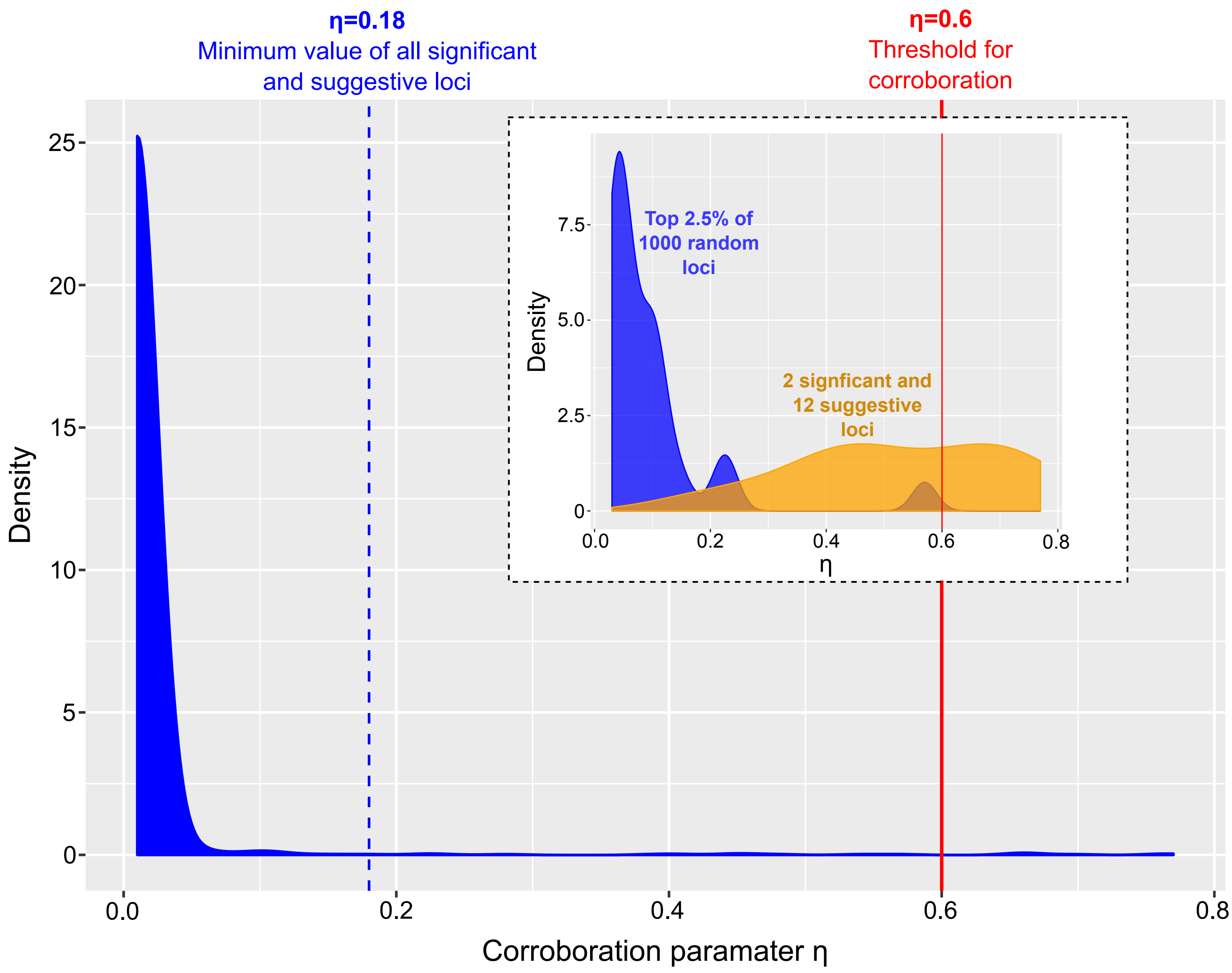

Supplementary Fig. 11. Distribution of corroboration values from 1,000 randomly selected loci and 14 loci significantly and suggestively associated with hibernation onset. The main plot shows the density of 1,014 variants across corroboration parameter (η) values after performing the ComPaSS-GWAS (see methods). The solid red vertical line denotes the threshold (η=0.6) at which variants were considered corroborated for significant association with hibernation onset. The dashed blue vertical line marks the lowest η-value observed among the 14 significant and suggestive loci. Insert shows density of the top 2.5% of 1,000 randomly selected loci (blue distribution) and the 14 significant and suggestive loci (orange distribution) across η.

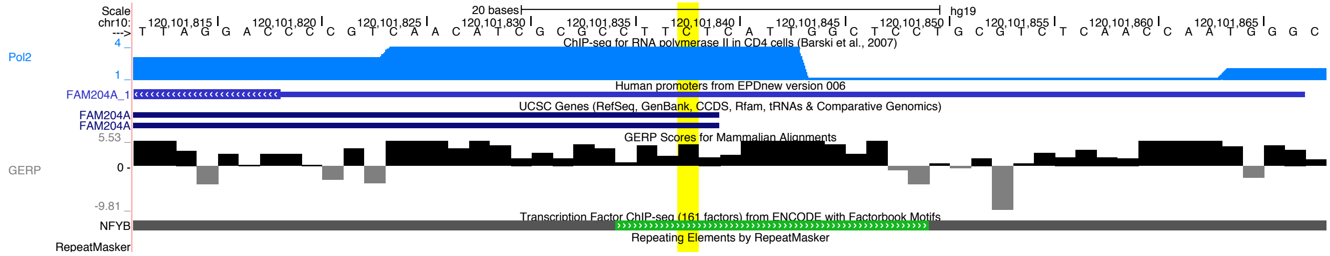

Supplementary Fig. 12. UCSC Genome Browser plot showing the homologous region for the SNP 1 putative causal variant on human hg19 Chr10. Yellow highlights the homologous SNP 1 putative causal variant (chr10:120,101,838; G>A, note that orientation is in reverse-complement on hg19). From top to bottom, the annotation tracks show: ChIP-seq peak for polymerase II, *FAM204A* promoter, *FAM204A* gene annotations, mammalian GERP++ scores and the ChIP-seq peak for *NFYB* transcription factor.

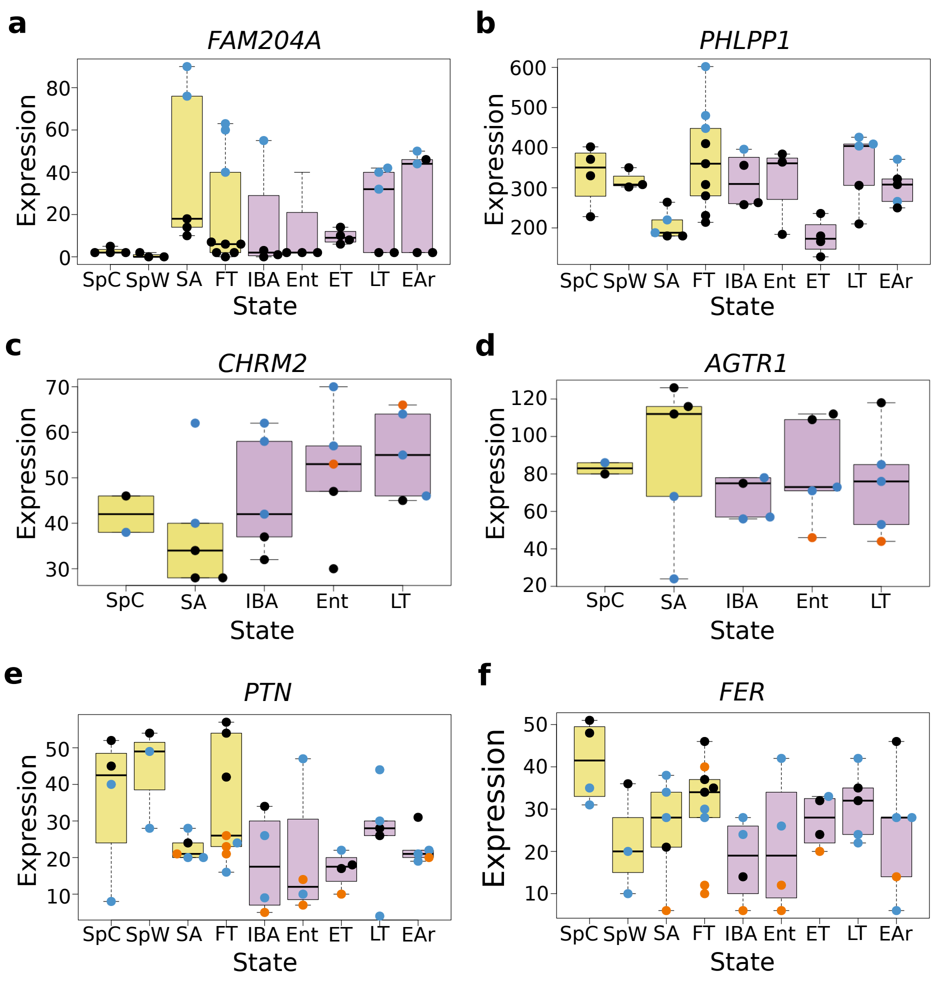

Supplementary Fig. 13. Box and whisker plots show cis-eGene and trans-eGene mRNA expression across physiological sample states. The colored boxes represent the region between the 25th and 75th percentiles, internal horizontal lines the median, outside horizontal lines the 100th percentile and unattached circles the outliers. Filled circles within each box represent the individual mRNA samples colored by genotype: black=major allele homozygous, blue=heterozygous and orange=minor allele homozygous. The mRNA expression counts, labeled on the y-axis, and sample states, labeled by abbreviation on x-axis, are taken from their original studies[1-2]. Abbreviations are as follows: spring cold, SpC; spring warm, SW; summer active, SA; fall transition, FT; interbout-aroused, IBA; entering torpor, Ent; early torpor, ET; late torpor, LT; early arousal, EAr. Shaded yellow boxes indicate physiological states from homeothermic and transitional portions of hibernator's year (spring-autumn), while purple shaded boxes are those from within deep hibernation. Distribution of SNP 1 genotypes and expression of its *cis*-eGene *FAM204A* (**a**) and *trans*-eGene *PHLPP1* (**b**) across sample states

in BAT (*n*=43). Distribution of SNP 5 genotypes and expression of its *cis*-eGene *CHRM2* (**c**) and *trans*-eGene *AGTR1* (**d**) in heart (*n*=22). Distribution of SNP 10 genotypes and expression of its *cis*-eGene *PTN* (**e**) and *trans*-eGene *FER* (**f**) in BAT (*n*=43).

Supplementary Tables

Supplementary Table 1. Pairwise FST estimates for the *K*=6 ADMIXTURE populations.

**WI LaX**	0.36				
**WI OK1**	0.36	0.45			
**WI OK2**	0.35	0.43	0.16		
**IL '10**	0.23	0.35	0.35	0.33	
**CO**	0.33	0.45	0.48	0.47	0.39
	**IL '06**	**WI LaX**	**WI OK1**	**WI OK2**	**IL '10**

See main text for population labeling.

Supplementary References

1 Grabek, K. R., Diniz Behn, C., Barsh, G. S., Hesselberth, J. R. & Martin, S. L. Enhanced stability and polyadenylation of select mRNAs support rapid thermogenesis in the brown fat of a hibernator. *eLife* **4**, doi:10.7554/eLife.04517 (2015).

2 Bogren, L. K., Grabek, K. R., Barsh, G. S. & Martin, S. L. Comparative tissue transcriptomics highlights dynamic differences among tissues but conserved metabolic transcript prioritization in preparation for arousal from torpor. *J Comp Physiol B* **187**, 735-748, doi:10.1007/s00360-017-1073-x (2017).
