# Supplementary material for: Genetic variation drives seasonal onset of hibernation in the 13-lined ground squirrel
Source: Commun Biol. 2019 Dec 20;2:478. doi: 10.1038/s42003-019-0719-5 (PMC6925185; doi:10.1038/s42003-019-0719-5)
Supplement: Supplementary file 2 — Description of additional supplementary items [file 42003_2019_719_MOESM2_ESM.docx]

**Description of additional supplementary items for**

Genetic variation drives seasonal onset of hibernation in the 13-lined ground squirrel

**Supplementary Data 1.** Details of the SpeTri2.0 draft assembly input and orientation of scaffolds into the final HiRise assembly

**Supplementary Data 2.** Summary of mapping results

For each ddRAD-seq library, details are supplied for the total number of sequencing reads, percent of reads mapped to the genome, percent of reads containing a single (BglII Mapped) or both (Target Mapped) restriction sites and the mean coverage for mapped reads containing both restriction sites.

**Supplementary Data 3.** Sample phenotypes

Biological, environmental and phenotypic records about the samples used in the heritability estimates and GWAS of hibernation onset, including year of monitoring (year), sex, age, date of datalogger surgery, date of placement into the hibernaculum, body mass at time of torpor onset, and date of first recorded torpor.

**Supplementary Data 4.** Heart *trans-*eQTL results

Table lists results for all *trans-*eqtl associations with p≤1X10^−4^

See Table 3 for SNP #

Tag ID is from the original Edge-tag datasets^1-2^

β is effect size estimated from quantile-normalized values

**Supplementary Data 5.** Skeletal muscle *trans-*eQTL results

Labeling is the same as in Supplementary Data 4

**Supplementary Data 6.** Liver *trans-*eQTL results

Labeling is the same as in Supplementary Data 4

**Supplementary Data 7.** BAT *trans-*eQTL results

Labeling is the same as in Supplementary Data 4

**Supplementary References**

1 Grabek, K. R., Diniz Behn, C., Barsh, G. S., Hesselberth, J. R. & Martin, S. L. Enhanced stability and polyadenylation of select mRNAs support rapid thermogenesis in the brown fat of a hibernator. *eLife* **4**, doi:10.7554/eLife.04517 (2015).

2 Bogren, L. K., Grabek, K. R., Barsh, G. S. & Martin, S. L. Comparative tissue transcriptomics highlights dynamic differences among tissues but conserved metabolic transcript prioritization in preparation for arousal from torpor. *J Comp Physiol B* **187**, 735-748, doi:10.1007/s00360-017-1073-x (2017).
