# Supplementary material for: Genetic variation drives seasonal onset of hibernation in the 13-lined ground squirrel
Source: Commun Biol. 2019 Dec 20;2:478. doi: 10.1038/s42003-019-0719-5 (PMC6925185; doi:10.1038/s42003-019-0719-5)
Supplement: Supplementary file 10 — Reporting Summary [file 42003_2019_719_MOESM10_ESM.pdf]

nature research

Corresponding author(s): Katharine Grabek

Last updated by author(s): Nov 13, 2019

Reporting Summary

Nature Research wishes to improve the reproducibility of the work that we publish. This form provides structure for consistency and transparency in reporting. For further information on Nature Research policies, see Authors & Referees and the Editorial Policy Checklist.

Statistics

For all statistical analyses, confirm that the following items are present in the figure legend, table legend, main text, or Methods section.

n/a	Confirmed	
☐	☒	The exact sample size (n) for each experimental group/condition, given as a discrete number and unit of measurement
☐	☒	A statement on whether measurements were taken from distinct samples or whether the same sample was measured repeatedly
☐	☒	The statistical test(s) used AND whether they are one- or two-sided<br>*Only common tests should be described solely by name; describe more complex techniques in the Methods section.*
☐	☒	A description of all covariates tested
☐	☒	A description of any assumptions or corrections, such as tests of normality and adjustment for multiple comparisons
☐	☒	A full description of the statistical parameters including central tendency (e.g. means) or other basic estimates (e.g. regression coefficient) AND variation (e.g. standard deviation) or associated estimates of uncertainty (e.g. confidence intervals)
☐	☒	For null hypothesis testing, the test statistic (e.g. F, t, r) with confidence intervals, effect sizes, degrees of freedom and P value noted<br>*Give P values as exact values whenever suitable.*
☐	☒	For Bayesian analysis, information on the choice of priors and Markov chain Monte Carlo settings
☒	☐	For hierarchical and complex designs, identification of the appropriate level for tests and full reporting of outcomes
☐	☒	Estimates of effect sizes (e.g. Cohen's d, Pearson's r), indicating how they were calculated

Our web collection on statistics for biologists contains articles on many of the points above.

Software and code

Policy information about availability of computer code

Data collection	Data was collected using commercially available software that was included with the data collection system, such as the iButton OneWireViewer software and the Meta-Meter™ Acquisition Program V2.4 for body temperature telemetry collection, as well as the Illumina Nextseq system suite v2.0.2 for collection of DNA sequence data.
Data analysis	Custom software used to lift over gene annotations as described in the methods is available at the following github repository: https://github.com/krgrabek/liftannot. All other data was processed and analyzed using publicly and commercially available software including: Chicago HiRise Scaffolder vNOV2015, SNAP v0.15.4, Bcl2fastq v2.5.14, Bwa v0.7.12, Sentieon v201611.01, Platypus v0.8.1, Samtools v1.3.1, GATK v3.7, ADMIXTURE v1.3.0, PRIMUS v1.9.0, VCFtools v0.1.12, KING v2.0, PLINK v1.9, SnpEff v4.3, Tomtom v5.0.5, FATHMM-MKL, R v3.5.1 and packages therein, including GENESIS v2.12.4, MCMCglmm v2.28, MatrixEQTL v2.2, WRShd v0.1 and ggmap v3.0.0.

For manuscripts utilizing custom algorithms or software that are central to the research but not yet described in published literature, software must be made available to editors/reviewers. We strongly encourage code deposition in a community repository (e.g. GitHub). See the Nature Research guidelines for submitting code & software for further information.

Data

Policy information about availability of data

All manuscripts must include a data availability statement. This statement should provide the following information, where applicable:

- Accession codes, unique identifiers, or web links for publicly available datasets
- A list of figures that have associated raw data
- A description of any restrictions on data availability

Sequencing data from the ddRAD-seq and whole-genome resequencing experiments were deposited at the NCBI Sequence Read Archive (SRA) under project accession PRJNA420609. Sequencing data from the HiRise Genome assembly experiment was deposited under the NCBI BioProject accession PRJNA420392. The datasets detailing 13-lined ground squirrel genetic variation and the hibernation onset GWAS summary statistics are available at the following Open Science

Framework repository: https://osf.io/a6v7w/?view_only=8d2841006be74207990f275de62d5436

Field-specific reporting

Please select the one below that is the best fit for your research. If you are not sure, read the appropriate sections before making your selection.

☒ Life sciences ☐ Behavioural & social sciences ☐ Ecological, evolutionary & environmental sciences

For a reference copy of the document with all sections, see nature.com/documents/nr-reporting-summary-flat.pdf

Life sciences study design

All studies must disclose on these points even when the disclosure is negative.

Sample size	This was an exploratory study performed on samples that were previously collected for use in other biochemical, proteomics and transcriptomics experiments. Sample size was therefore limited to the availability of samples.
Data exclusions	Genetic variation data (i.e. SNPs) were excluded if they did not pass QC filtering by individual variant callers, were not identified across all three variant callers, were < 95% concordant among the three variant callers, exhibited excessive coverage (>4x mean coverage) or heterozygosity or were missing in greater than 10% of the samples.
Replication	The main findings were not replicated independently, because, to our knowledge, there are no other large populations of 13-lined ground squirrels for which both genetic variation data and phenotype measurements of torpor onset exist. However, we used an alternative published method for independent replication to corroborate the variants identified as significantly or suggestively associated with the onset of hibernation. We also replicated the genotype calls at the most-significant locus (SNP 1) in 12 samples using whole-genome sequencing.
Randomization	The samples were not allocated into groups, because the phenotype (date of first torpor) was measured on a continuous scale. However, to identify non-genetic factors that could affect the date of first torpor, we applied a linear regression using variables available from records about the squirrels. We then pruned factors using step-wise regression until we identified a minimum set of variables that did not significantly reduce the adjusted R-squared value from the initial model, yet also returned a low AIC value. These were input as our fixed effects in the linear mixed model.
Blinding	Blinding was not relevant to this study, because this was not a research trial designed to test the effect of a medical treatment.

Reporting for specific materials, systems and methods

We require information from authors about some types of materials, experimental systems and methods used in many studies. Here, indicate whether each material, system or method listed is relevant to your study. If you are not sure if a list item applies to your research, read the appropriate section before selecting a response.

Materials & experimental systems

n/a	Involved in the study	
☒	☐	Antibodies
☒	☐	Eukaryotic cell lines
☒	☐	Palaeontology
☐	☒	Animals and other organisms
☒	☐	Human research participants
☒	☐	Clinical data

Methods

n/a	Involved in the study	
☒	☐	ChIP-seq
☒	☐	Flow cytometry
☒	☐	MRI-based neuroimaging

Animals and other organisms

Policy information about studies involving animals; ARRIVE guidelines recommended for reporting animal research

Laboratory animals	One-hundred-and-thirty colony-bred 13-lined ground squirrels (Ictidomys tridecemlineatus; 68 females and 62 males) were obtained from the University of Wisconsin, Oshkosh in the summers of 2007—2010. These included 73 juveniles naïve to hibernation in the year of study, and 57 adults with at least one year of hibernation. While most from the colony were bred from squirrels originally wild-trapped in northeastern Wisconsin (in and around Oshkosh), several of those obtained from the Oshkosh colony in 2010 were actually bred from either a single or both parents wild-trapped in far western Wisconsin, more than 100 miles away (in and around La Crosse, WI). However, records to identify these specific squirrels were not always maintained.
Wild animals	Seventeen 13-lined ground squirrels (nine females and eight males; ages unknown), wild-trapped in different locales around central Illinois, were obtained from a commercial supplier (TLS Research, Bloomington, IL) in the summers of 2006 and 2010. Six 13-lined ground squirrels (three females, three males; ages unknown) were wild-trapped in the summers of 2006 and 2009 in Elbert County and Larimer County, Colorado.

Field-collected samples	Upon arrival, animals were housed individually in rodent cages under standard laboratory conditions (20±2°C and 14:10 light-dark cycle, fed cat chow supplemented with sunflower seeds ad libitum). In late August or early September, animals not yet euthanized for tissue collection were surgically implanted with an intraperitoneal datalogger (iButton, Embedded Data Systems) and/or a radiotelemeter (VM-FH disks; Mini Mitter, Sunriver, OR) for remote body temperature (Tb) monitoring until tissue collection. The dataloggers recorded Tb±0.5°C every 20, 30 or 60 min, while the radiotelemeters transmitted Tb±0.5°C every 20 sec. In late September or early October, the squirrels were moved to the hibernaculum to facilitate hibernation. The temperature was lowered stepwise over a two-week period to 4°C. Food was removed as animals became torpid.
Ethics oversight	All animal use was approved by the University of Colorado, Anschutz Medical Campus Animal Care and Use Committee.

Note that full information on the approval of the study protocol must also be provided in the manuscript.
